# Decision-Making in Virtual Reality Sports Games Explained via the Lens of Extended Planned Behavior Theory

**DOI:** 10.3390/ijerph20010592

**Published:** 2022-12-29

**Authors:** Bo-Hyun Seong, Chang-Yu Hong

**Affiliations:** 1 Chungbuk Research Institute, Cheongju-si 28517, Republic of Korea; 2 Division of Global and Interdisciplinary Studies, Pukyong National University, Busan 48513, Republic of Korea

**Keywords:** virtual reality sports game, perceived interactivity, extended theory of planned behavior, COVID-19

## Abstract

This study aims to determine whether the effect of interactivity on participation in virtual sports has risen because of the COVID-19 pandemic and if there is a difference in decision-making. The significance of social factors may be highlighted even more as a rationale for using virtual reality (VR) sports apps throughout the prolonged COVID-19 epidemic. A model was built based on the literature to describe the intention to participate in VR sports, and eight associated hypotheses were established. A sample of 301 submissions from a user poll on Korea’s cycling information sharing website was used for our analysis. SPSS 23.0 (IBM, Armonk, NY, USA) and AMOS 18.0 (IBM, Armonk, NY, USA) were used to validate Hypotheses 1–8 using a multigroup structural equation model (SEM) analysis and multigroup analysis. Although some hypotheses were not validated, the impact of perceived interaction presented as an extra variable altered based on the group participating before and after the COVID-19 epidemic, and the study’s goal was achieved. Given that information technology has evolved by overcoming physical space and socio-cultural constraints to create a society that connects people, the importance of online interaction, such as networking and competition between users, will be emphasized in the VR sports field in the future.

## 1. Introduction

The worldwide pandemic of COVID-19 has caused a scarcity of in-person visits and cultural exchanges between nations since early 2020, and most governments have established social distancing prevention laws [1,2]. People are spending more time at home and are embracing new lifestyle trends such as telecommuting, online learning, non-face-to-face meetings, and online shopping. Behavior that has led to the enjoyment of leisure activities via engagement with other people has also reduced the amount of interpersonal contact maintained through online platforms and smart devices. Not long ago, digital technology, which has been used primarily to measure, analyze, or broadcast athletes’ performance in the real world [3], has led to commercial success in sports applications seeking a more holistic approach to health and play by providing several functions to the general user [4,5].

Most previous studies stressed that training effectiveness has a significant impact on the usage of sports applications. Consequently, sports applications on smart devices have been recognized as helpful training instruments for increasing physical performance [6]. Although mechanisms can be likened to health benefits, the goal of exercise is to improve performance. In this context, customers appreciate sports applications that enable them to tailor their training to their specific needs [7]. Virtual reality (VR) apps are beneficial in training settings because they enable users to “receive feedback on performance and practice specific skills” [8]. VR systems provide more satisfaction and more consistent fitness routines during exercise [9], which is why they are often associated with healthier living.

Nevertheless, the significance of social factors may be highlighted even more as a rationale for using VR sports apps throughout the extended COVID-19 epidemic. Although a person fundamentally desires to know the extent of their abilities, the measures caused by social distancing fundamentally block the comparison of exercise performance through interaction with others offline. The VR setting can overcome limitations imposed by physical space, and it is a powerful component that maintains the continuity of involvement by encouraging users to communicate and collaborate [8,10]. Competitive factors, such as ranking, improve users’ athletic ability [11]. This strategy is vital for promoting the use of sports applications [12].

Sports digitization is about the connection between players. Zwift, an online cycling game with 16 million members worldwide as of early 2020 [5,13], is a good illustration of this. Although the primary goal of using a VR sports application in a general context may be to improve performance, in the wake of the COVID-19 epidemic, it is also essential to consider the benefits of using a VR sports app to connect with others. There are several benefits to participating in an activity with others rather than engaging in it alone, such as fostering happy feelings and a sense of absorption [14,15]. Because human behavior has been thoroughly studied in numerous sectors, this study attempts to broaden the idea of planned behavior to examine the impact of interaction on VR sports participation [16].

The VR sports industry and community have recently focused on golf, baseball, and cycling. Cycling is a representative sport that attracts many fans worldwide. Bicycles were Korea’s fifth most popular sport, behind trail walking, mountain climbing, bodybuilding, yoga, pilates, and golf [17]. We want to examine why so many individuals enjoy cycling. It might be relatively easy to enjoy in Korean society because of the expansion of infrastructure, such as the creation of bicycle roads, which began in earnest in the early 2000s. It could also be a reason to keep encouraging growth because it fits in with the global eco-friendly mindset by reducing traffic and carbon emissions from daily travel and securing fun activities.

This research topic has been conducted to provide an academic explanation for the decision-making process that leads to participation in VR sports [4,16,18,19,20,21]. Related research is scarce because few researchers have attempted it to date. Thus, we endeavor to create the first model to our knowledge that describes the decision-making path of participation in virtual bicycle sports as leisure activities by expanding the theory of planned behavior (TPB) with perceived interactivity as an additional factor.

Furthermore, just as social media usage for communication has expanded fast because of the COVID-19 worldwide pandemic, VR sports produced using modern innovative information technology may offer a greater incentive to pursue online interaction such as connection and competitiveness. Consequently, we want to determine whether there is a difference in the decision-making process of engaging in VR sports by splitting the participants into two groups: those who used Zwift before and after the outbreak of COVID-19. Given that information technology has progressed by transcending physical space and socio-cultural limits and aiming for a society that links people, online conversations on user engagement through mobile networks can explain the decision-making process in VR sports.

## 2. Conceptual Note and Literature Review

### 2.1. VR Sports

Modern society’s production and consumption patterns have shifted because of technological development and the rapid rise of the information and communications technology (ICT) industry [22]. For instance, digitization alters how individuals participate in sports [23]. In sports, digital technology has primarily been used to measure, analyze, or broadcast the performance of professional athletes [3], and recently numerous sophisticated VR sports applications have been developed for baseball, cycling, fishing, tennis, badminton, swimming, and climbing [4]. Despite the necessity for digitizing sports to remain a crucial feature of life in the event of contagious diseases, few sports have attained widespread popularity [24].

The ultimate goal of sports digitization is to combine online and offline experiences. When the online and offline markets are mutually interdependent and complementary, they can contribute to the growth of the sports event industry. As of early 2020, relevant representative examples include the online cycling game “Zwift” [13], which has 16 million users worldwide, and screen golf, which has been commercially successful in Korea [4]. Because screen golf is widely recognized for its optimistic outlook on growth sustainability and the social and economic importance of related corporations entering the Northeast Asian market, many academic discussions on the decision-making process leading to participation have taken place [4,16,19,20].

However, screen golf has a limitation as a sports activity because it does not meet the sports standard [5,25] of having a large following that is not limited to a specific region. Screen golf is a tool for improving personal skills. However, it is insufficient as a sports platform that enables users worldwide to interact in real-time in VR anytime by overcoming space and time constraints. Zwift, in contrast, meets the sports standards proposed by previous researchers because it has a large number of users who voluntarily participate in various countries [25], and competition is governed by institutionalized rules on physical technology [26]. 

The reigning Olympic champion and three Tour de France winners took part in the first “Virtual Tour de France” in July 2020, and the race was broadcast in over 130 countries, demonstrating that virtual cycling platforms such as Zwift have reached the pinnacle of sport [27]. Non-professionals participated in the event through mass participation races held on the same virtual courses as professionals [27].

Zwift’s unique advantage as a VR sport is that it has achieved technological development that satisfies user technology improvement and enabled interactions that can be connected in real-time to compete with users worldwide. As the saying goes, “A universal case of interesting activity is being with others” [28]. Experiencing and interacting with others rather than participating alone in a particular activity helps positive emotions and immersion [14,15]. Because of the COVID-19 epidemic, people appear to enjoy spending time alone at home. However, in VR, people seek pleasure through interactions that enable them to experience and compete with other individuals pursuing public goals. Accordingly, Zwift is a powerful platform because it enables users to conduct previously unexplored research in the field of VR sports from the perspective of user interaction.

### 2.2. Extended TPB

**Figure 1:**
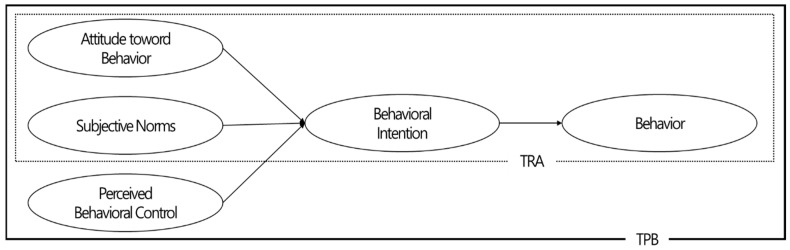
Theory of Reasoned Action (TRA) and Theory of Planned Behavior (TPB). Note: Theory of Reasoned Action (TRA), Theory of Planned Behavior (TPB). Source: Ajzen & Fishbein (1975). A Bayesian analysis of attribution processes; Ajzen (1988). Attitudes personality and behavior. Buckingham, UK: Open University Press.

Social psychologists have long been fascinated by the process of human behavior. A person’s attitude has been discussed extensively among the various psychological variables because it is a reliable indicator of how humans behave [29]. Based on empirical studies, various factors can affect the strength and stability of attitudes. Research to add variables that can increase the accuracy of behavior prediction in addition to attitudes has begun in earnest. Among the proposed theories in this context, rational behavior theory [30,31] and theory of planned behavior (TPB) [32,33] are widely accepted and used (Figure 1).

Behavior is explained by rational behavior theory through behavioral intention. The intention to perform a specific action can be explained as the direct influencing factor of behavior [34]. Consequently, behavioral intention is defined as a medium that mediates the effects of attitudes and subjective norms on behavior [35]. However, rational behavior theory is still criticized for its limitations because it is based on the fundamental premise that all actions can be fully controlled by the individual [36,37]. Thus, planning behavior theory has been proposed to add perceived control to the extent to which specific behavioral performance is under individual willful control [38] and is used as a practical theoretical framework in various fields to explain how human behavior occurs [39,40,41,42,43,44,45,46].

However, because of the low explanatory power of three independent variables—attitude of planned behavior theory, subjective norms, and perceived behavior control—many researchers agree that a continuous search for additional variables affecting behavior should be performed [36,37,47,48]. Ref. [33], who proposed the TPB, also explained that it was fundamentally an open theory by extending the path to explain behavioral intent via additional variables. Accordingly, until recently, numerous studies were deemed to have successfully expanded the TPB by adding variables deemed crucial to a particular setting [16,49,50,51,52,53].

The following explains why the debate over the expansion of the TPB continues. From the standpoint of activism, human behavior is influenced by the ever-changing social and physical environment. The recent COVID-19 pandemic is an example of a social environment change that significantly impacts human behavior. Most countries have implemented social distancing as a major action immunity strategy for epidemic control [54,55,56,57]. Various events and gatherings where a large number of people gathered were controlled, and intensive social distancing measures were implemented to encourage people not to go out. However, humans are social animals. It is human nature to want to connect with and communicate with others. Human instincts are being expressed in search of some possible alternative, just as the use of social media for communication has rapidly increased because it has become impossible to face others in everyday life.

Furthermore, Web 2.0 platforms are enabling online interaction beyond communication. For example, VR sports based on information technology have a higher proclivity to participate in online interactions such as user connection and competition—experiencing and interacting with others, rather than participating alone in a specific activity, promotes positive emotions and immersion [14,15]. It is possible that using VR sports applications in a general setting is intended to improve performance. Consequently, it is important to discuss how participation in VR sports applications can be used as a tool for interacting with other people during a massive shift in the surrounding environment brought on by the COVID-19 pandemic.

Until date, this study team has used a variety of models to describe the process of adopting digital sports. The process of using screen golf was explained using the technology readiness and acceptance model (TRAM) [4], and the difference in the decision-making process between screen golf and screen baseball users was explained using the technology acceptance model (TAM) [57]. Based on the findings of this study, the research team intends to improve and maximize the usage of the extended theory of planned behavior model (ETPB) as part of a research series on digital sports.

### 2.3. Perceived Interactivity in VR Sports

Social interactions take place primarily within social organizations that adhere to particular norms. However, they can also occur in free and transient social relationships (communita) formed through participation in festivals, leisure, tourism, and sports [58]. Emotional expression and behavior formed in play-for-play temporary communities [59] create an atmosphere that can be fused and assimilated into the collective environment [58,60]. Individuals with similar interests gather simultaneously and space and form a consensus by doing specific activities suggested by service providers, which is why exchanges between spectators can be active in performance venues, sports stadiums, and festivals [58,61,62,63,64].

Extensive interaction-related research has been conducted in the new media sector, as evidenced by the digital convergence phenomena [65]. Some researchers [65,66] found that interaction between users is one of the most important ways to distinguish new media from old media. increasing interaction enhances the efficacy of most web-based formats and content [67]. The idea that it is critical to quantify the degree to which a particular environment is seen as interactive has gained widespread favor [67,68,69,70]. The concept of perceived interaction in the sphere of new media has significant ramifications for the subject of sports digitization.

Factors such as competitiveness and cooperation, notions related to interaction in sports, have a favorable effect on participants in both online and offline settings [71]. Rankings are one of the primary tactics for promoting sports applications [11] and can enhance users’ athletic skills [12]. People compare their skills to others through objective or subjective evaluations; this social comparison drives them to do harder and better, close the performance gap with others, and outperform others [72].

**Figure 2:**
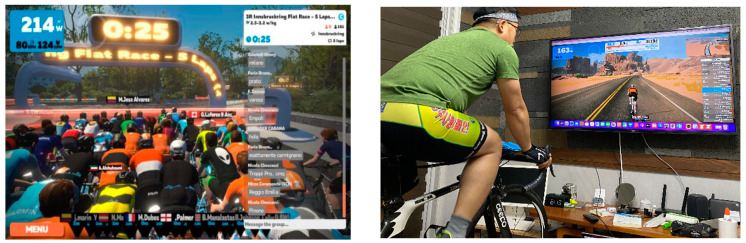
Zwift screen capture (**Left**); researchers playing Zwift application (**Right**). Source: https://blog.cyclestore.co.uk/zwift-racing-5-top-tips/ (Accessed on 5 March. 2022) (**Left**), a researcher’s photograph (**Right**).

Sports games should provide opportunities for players to compete, collaborate, and interconnect to foster interaction, and they should be designed to increase enjoyment through engagement with others [73,74]. Sanz, Multon and Lécuyer [75] highlighted the importance of discussing interactions with other users during the training process of athletes using VR platforms. Furthermore, during the COVID-19 epidemic, ref. [5] interviewed pro-cyclists using the VR sports platform Zwift, indicating that users valued the ability to engage with other digitally mediated users (Figure 2).

Interaction between users should be considered when discussing the enjoyment of VR sports, although relevant research is scarce. Previous research has concentrated on the effectiveness of training because of the usage of sports applications, primarily highlighting its significance as an effective, tailored training tool for enhancing physical performance [6,7]. The COVID-19 pandemic’s quarantine strategy has limited outdoor sporting activities and communication. However, by facilitating interaction between users, VR environments can overcome physical space constraints and be highly influential in sustaining participation in particular activities [8,10], i.e., the role of decision-making in using VR sports applications during the COVID-19 pandemic.

## 3. Research Design and Methodology

### 3.1. Research Model and Hypotheses

**Figure 3:**
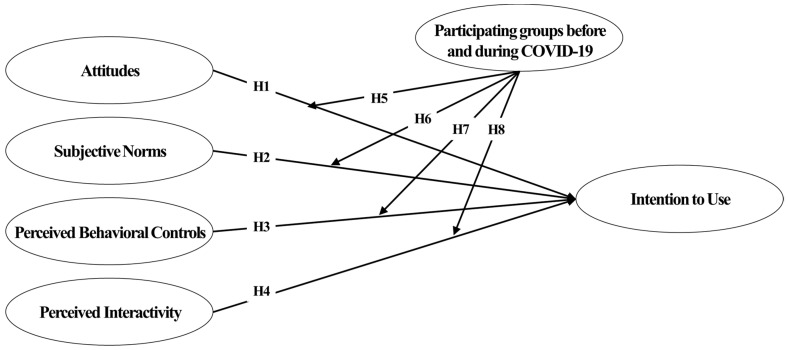
Research model.

A model was built based on literature research to describe the intention to participate in VR sports, and eight associated hypotheses were established (Figure 3). Hypotheses 1–4 describe the influence link between independent factors and participation intention, stating that personal attitude, subjective norms, perceived behavioral control, and perceived interaction all impact participation intention. Hypotheses 1–3 are based on the overall findings of the previously stated theory of planned behavior (TPB) Study [33,39,41,43,46,53,76,77].

**Hypothesis 1.**  *Attitudes will positively affect the intention to use VR sports.*

**Hypothesis 2.**  *Subjective norms will positively affect the intention to use VR sports.*

**Hypothesis 3.**  *Perceived behavioral controls will positively affect the intention to use VR sports.*

Hypothesis 4 describes how perceived interaction, when used as an additional variable, has a positive effect on participation intention and establishes a previously discussed debate [5,11,71,75].

**Hypothesis 4.**  *Perceived interactivity will positively affect the intention to use VR sports.*

Hypotheses 5–8 were developed to explain how the COVID-19 epidemic influences the interaction between attitudes, subjective norms, perceived behavioral control, structural tourism restrictions, and participation intentions. No study directly verified the influence relationship between variables set in the hypothesis. Nevertheless, after COVID-19, the relevance of social factors as a rationale for using VR sports applications is further emphasized because social distancing methods prevent comparison of exercise accomplishment through offline connections with others. VR environments can transcend physical space limits and have a favorable effect on participation in specific activities by increasing user interaction [8,10]; their influence would have increased after COVID-19. For Hypotheses 5–8, the influence relationship between variables varies depending on whether the group participated before or during COVID-19.

**Hypothesis 5.**  *The effect of attitude on intention varies depending on whether the group participated before or during COVID-19.*

**Hypothesis 6.**  *The effect of subjective norms on intention varies depending on whether the group participated before or during COVID-19.*

**Hypothesis 7.**  *The effect of perceived behavioral control on intention varies depending on whether the group participated before or during COVID-19.*

**Hypothesis 8.**  *The effect of perceived interactivity on intention varies depending on whether the group participated before or during COVID-19.*

### 3.2. Data Collection and Analytical Design

A questionnaire with 30 questions was used to measure six variables from the hypotheses and demographic information about respondents. Previous research was used to develop the measurement items. Previous studies on planning behavior theory were used to construct assessments of attitudes, subjective norms, perceived behavioral control, and participation intention [33,39,41,46,76,77]. Regarding perceived interaction, a theoretical survey in a behavior-related study was conducted on members of DOSSA (https://corearoadbike.com/) (accessed on 10 March 2022), where around 250,000 online bikers in Korea joined and shared information. The participants were chosen from among the members who use Zwift, and the test occurred between 1 and 15 September of the following year (2021).

Based on the survey, 301 valid samples were selected for the final analysis, omitting questionnaires determined to have been answered insincerely, and data analysis was conducted using SPSS 23 and AMOS 18.0. Frequency analysis and K-means clustering analysis were used to determine the general features of the respondents, and the validity of each measurement variable was validated using confirmatory factor analysis, correlation analysis, and reliability analysis tools. Finally, a structural equation model (SEM) is used to verify Hypotheses 1–4, and multigroup analysis with SEM is used to verify Hypotheses 5–8.

## 4. Results

### 4.1. Respondents’ Demographic Characteristics

A total of 301 valid samples were analyzed to identify demographic variables such as gender, residence, age, occupation, average household income, and academic background (Table 1). Males (52.2%) outnumbered females (47.8%), and those aged 30s (76.4%) outnumbered those in their 20s (14.6%) and 40s (6.0%) and 50s (2.0%). In addition, 84.4% answered that households earn more than 5 million won a month. Seoul (24.3%), which was one of the survey sites, showed the highest rate of residence and relatively large percentages of people residents from nearby area such as Gyeonggi-do (17.9%), Incheon (10.6%), Busan (10.0%).

The results of the survey judged that there was no problem in representing the gender and regional population distribution presented in the demographics released by the Ministry of Public Administration and Security (https://www.mois.go.kr/eng/a01/engMain.do) (accessed on 25 January 2022) as of September 2021. The fact that college graduates or higher, office worker, monthly income per household of more than 5 million won, and the high proportion of people in their 30s were also judged to be acceptable as characteristics of the demand group that uses both cycle and VR Sports Zwift.

### 4.2. Confirmatory Factor Analysis

The fit index of the measurement model was χ^2^/df = 1.410 (χ^2^ = 332.653, df = 236), RMR = 0.028, RMSEA = 0.037, GFI = 0.917, NFI = 0.905, RFI = 0.889, IFI = 0.970, TLI = 0.965, and CFI = 0.970, based on the confirmatory factor analysis for the validity verification of the measurement items. The recommended fit index criteria are χ^2^/df ≤ 3, RMSEA ≤ 0.08, RMR ≤ 0.05, and GFI, NFI, TLI, CFI = 0.9 or higher. However, one of the survey items used to calculate interactivity was 0.390, which was removed because it did not fulfill the requirement (0.5–0.95).

After removing survey questions that were not applicable, a new round of confirmatory factor analysis was conducted. Based on the findings, the majority of the fit indices of the measurement model belonged to the category comprised of the recommended criteria (χ^2^/df = 1.394 (χ2 = 299.627, df = 215), RMR = 0.026, RMSEA = 0.036, GFI = 0.921, NFI = 0.912, RFI = 0.896, IFI = 0.973, TLI = 0.968, and CFI = 0.973). Convergent validity was demonstrated because the scores on each measurement item of construct reliability (CR) (higher than 1.965, *p* < 0.05), standardized factor loading (0.5–0.95), CR (recommended range—higher than 0.7), and average variance extracted (AVE) (recommended range—higher than 0.5) were all higher than the reference value (Table 2).

In contrast, based on the correlation analysis between measurement items, the correlation coefficient between measurement items in Table 3 appeared to be between 0.122 and 0.789, which is within the recommended level of 0.85 [78]. The average dispersion extraction index (AVE) of each measurement item was more valid than the squared value of the correlation coefficient [79].

### 4.3. Hypothesis Testing

(1) Verification of Research Hypotheses 1–4

We examined the parameter estimates and the goodness-of-fit of the SEM to determine whether the hypothesized model was a good fit for the collected data. This good fit was confirmed by the SEM’s fit indices, as follows: 2/df = 1.394 (2 = 299.627, df = 215), RMR = 0.026, RMSEA = 0.036, GFI = 0.921, NFI = 0.912, RFI = 0.896, IFI = 0.973, TLI = 0.968, and CFI = 0.973. Therefore, the relationships between the five conceptions were investigated to test the four hypotheses (Table 4).

Following hypothesis verification, attitude, perceived behavioral control, and perceived interaction all had a substantial effect on the intention to use, validating Hypotheses 1, 3, and 4. In contrast, the association between subjective norms and intention to use was not significant. Hence, Hypothesis 2 was not supported. The results of the hypothesis verification revealed that the individual’s attitude has a relatively significant effect, consistent with the findings of earlier studies [80,81] that explain the first key drivers of behavioral intent on attitude.

The Hypothesis 4 that a relationship exists between perceived interaction and intention to use is supported, and the significance of the hypothesis verification result can be emphasized as a variable for the planned behavior theory’s extension. The influence of perceived behavioral control, in contrast, was the lowest among the influence relationships indicated by the hypothesis, which is significant because of specific changes from previous studies [53,76,82,83].

(2) Verification of Hypotheses 5–8

Hypotheses 5–8 investigate whether the influence relationship between the independent variables (subjective norm, perceived behavioral control, and perceived interactivity) and the dependent variable (intention to use) varies depending on the COVID-19 component. For this hypothesis verification, a multigroup study was performed, dividing the group that used Zwift (n = 140) during the COVID-19 pandemic from the group that used it (n = 161) before the COVID-19 pandemic. A multigroup analysis is a three-step procedure that starts with a multigroup confirmatory factor analysis and concludes with a multigroup SEM analysis. The multigroup confirmatory factor analysis was intended to verify that the measurement scales in this study had measurement uniformity in two groups, which was determined by comparing the difference value between non-constrained and constrained models (χ^2^, df) with a chi-square distribution table.

In the context of this study, it was necessary to verify that each group—users who started during the COVID-19 pandemic and users who started before the COVID-19 pandemic—understood the concepts of composition in the survey similarly, a pre-validation process for multigroup path analysis. The results of the measurement homogeneity analysis reveal a difference between the non-constrained model and the constrained model of Δχ^2^ = 13.27 and Δdf = 18, which are presented as Δχ2 = 28.87 when *p* = 0.5 and df = 18. The difference of χ^2^ between non-constrained and constrained models is not statistically significant because they are smaller than the values presented in the χ^2^ distribution table. Consequently, there is no issue with measurement tools concerning measurement uniformity (Table 5).

Concerning this study’s idea of composition, a multigroup route analysis was conducted because the level of measurement invariance was not problematic. Consequently, differences in the relationships between perceived interaction and intention to use and between perceived behavioral control and intention to use were discovered (Table 6). Only in the group who used Zwift before the COVID-19 epidemic was the relationship between perceived behavioral control and intention to use significant. However, only in the group using Zwift during the COVID-19 pandemic was the relationship between perceived interactivity and intention to use significant.

**Figure 4:**
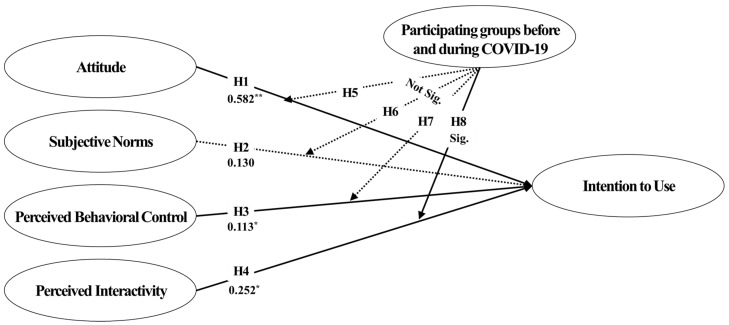
Research model and hypothesis verification results. Notes: *: <0.05 **: <0.01.

Even if only one group is a significant path, the difference between paths may be significant, and even if both groups are not significant, a significant difference between paths may exist. A multigroup SEM analysis was used to establish the statistical significance of all pathways. Consequently, the difference in χ^2^ was greater than 3.84 (*p* = 0.05), equivalent to df = 1 in the χ^2^ distribution table, which was statistically significant in the association between perceived interactivity and intention to use. Therefore, based on the modulation effect verification (Table 7), Hypothesis 8 was supported (Figure 4).

## 5. Discussions

This study used an expanded version of the planned behavior theory to explain the decision-making process of a cyclist who selects the VR sports application Zwift. Based on the literature review, four hypotheses were established concerning the influence of attitude, subjective norm, perceived behavioral control, and perceived interaction on intention to use, and four additional hypotheses concerning the role of the COVID-19 pandemic as a moderating variable. Although some hypotheses were not validated by the analysis, the research goal was achieved based on the effect of perceived interactivity as an additional variable and the verification of differences between groups relative to the COVID-19 pandemic.

Furthermore, our hypothesis set was corroborated using data acquired from a poll of 301 Zwift users. The influence of attitude, perceived behavioral control, and perceived interaction on intention to use were significant across all eight hypotheses, while the moderating effect of the COVID-19 pandemic was significant only in the link between perceived interaction and intention to use.

First, the variable with the most significant influence on the intention to use Zwift was “attitude”, with a significant influence when compared with other variables. The more positive cycling users are, the higher the involvement, so technology-oriented promotional professionals must promote the benefits of VR applications. Zwift, as a VR sports application, does not fall short as a tool for improving cycling skills, and it has the added benefit of saving time and money because it can be enjoyed at any time. Furthermore, discontent and concern about sports activity limitations caused by weather circumstances such as summer heat, winter cold, rain, and snow might be alleviated.

That users can work out with other bikers and experience competitive interaction, as validated in this study, is an advantage that may also be emphasized more in the era of the untapped New Normal because they can do it together. Efforts by business marketers to emphasize the benefits of motivation to increase involvement are required.

Existing studies had a substantially lesser influence on subjective standards than on other factors. The second hypothesis that subjective norms have a favorable influence on intention to use was not validated. The opinions of critical persons influencing decision-making can be expressed in a weighted value at the initial start of cycling. However, it can be deduced that aspects that might affect an individual’s will, such as attitude, perceived behavioral control, and perceived interaction, are reflected in the process of selecting VR sports applications, which can be called training assistance, after the start of the cycle. According to several Planned Behavior Theory studies [33,40,47,84], subjective standards have a relatively low influence on intention when compared with other variables. Regarding “participation”, the relationship between subjective norm and intention is not relevant when motivation about the purpose and expense of exercise training is accompanied, as in screen golf [4].

The moderating effect, in contrast, verifies that the influence link between the proposed independent and dependent variables in the study model would change depending on the group (the groups using Zwift during and before the COVID-19 pandemic). The group that began using Zwift during the COVID-19 pandemic had a significant association between perceived interaction and intention to use. Statistical significance for the difference between groups in the path was also confirmed. The findings indicate that social interaction can be a significant element in selecting VR sports applications. In contrast, the social distance-making policy resulting from the COVID-19 pandemic has been long-lasting. Furthermore, the association between perceived behavioral control and intention to use was only significant in the group that began using Zwift during the COVID-19 pandemic. However, the statistical significance of the difference between the path groups was not confirmed.

By examining the decision-making process based on the option, it is possible to conclude that the influence of the opinions of those around them has weakened at the stage of selecting the use of VR sports applications for individuals who already enjoy cycling. The opinions of those around them can influence the use of VR sports applications.

Many previous studies have validated the influence relationship between perceived interaction and intention to use. The TPB has concentrated on improving the explanation of the process of specific conduct by adding variables around attitude. Regarding perceived behavioral control, which was stressed by the idea of planned behavior, the theory had a relatively small influence. These findings highlight the importance of the extended planned behavior theory in explaining the process of individual actions by including factors that can reflect specific scenarios. Walker (2010) noted that social flow experienced with others rather than alone has a favorable effect on enjoyment and positive emotions regarding boosting engagement in specific activities. Even though they performed specific tasks with others, they found that interaction commitment with an emotional connection was superior to individual commitment, a condition of emotional separation.

The advancement of information technology has enabled interactivity-oriented communication that transcends spatial and temporal boundaries. Interactivity encompasses all actions of exchange between human individuals, human groups, and things in a particular setting. The extent to which interaction in VR impacts the structure and content of the environment mediated by information technology can be examined.

Humans have an instinctive urge to communicate with others. The topic of the so-called “Fourth Industrial Revolution Industry 4.0”, which is represented by the commercialization of spatial artificial intelligence, human robots, and unmanned vehicles via information technology development, or the hyper-connected society, is the development of new technological achievements and innovative communication methods, and the development of human communication methods. It is the result of increased cognitive ability and wider world recognition.

Because Zwift and screen golf can overcome major obstacles such as weather, time, and accessibility, most individuals who enjoy the event online also actively participate in the event outdoors. Screen golf is propelling the expansion of the overall golf industry by addressing various obstacles such as weather, accessibility, and cost. One of its primary functions will be to improve individual ability through golf training in VR; it will evolve into a platform that can reflect the performance of the actual exercise in real-time in the VR program in the training curriculum. It is feasible to evolve screen golf into a form distinct from sports games or active video games, and it can be popularized because of the benefits consumers seek through sports participation.

Social psychologists have long been interested in describing the process of human action. In empirical studies, individual attitude was once thought to be the most dependable psychological characteristic in predicting behavior. The most generally used theories regarding human action are the theory of reasoned action [85] and the TPB [33]. However, the strength and stability of attitudes are insufficient to predict action because they are impacted by several events [29]. Consequently, various variables to boost behavior prediction explanation were investigated in addition to attitude. 

COVID-19 Global Pandemic has prohibited international contact since early 2020, and most countries have enacted social distance prevention policies [1,2]. The amount of time spent at home has increased, as has telecommuting, online learning, non-face-to-face meetings, and internet shopping. Behavior that resulted in the enjoyment of leisure activities through engagement with people also reduced interpersonal contact via online platforms. Not long ago, digital technology, which was primarily used to measure, analyze, or broadcast athletes’ performance in the real world, led to commercial success in sports applications seeking a more holistic approach to health and play by providing several functions to the general user [4,5].

The purpose of using VR sports applications may be to optimize performance. However, at this point, as the world is experiencing significant environmental change because of the COVID-19 pandemic, it is necessary to discuss the benefits of VR sports applications as a tool for interaction with others. Because the COVID-19 global pandemic prevented individuals from interacting in ordinary life, social media use for communication skyrocketed. Information technology-based VR sports may be more likely to facilitate online interactions like user connection. However, similar conversations were held in the study to explain the decision-making process leading to VR sports involvement [4,16,18,19,20,21,42]. Because information technology has advanced by breaking down geographical and cultural barriers to create a society that brings people together, online debates about user engagement can help to make more people aware of how they can participate in VR sports.

Given that there should be a diverse range of participants from all over the world, one of the main characteristics of VR sports, it is critical to secure factors that encourage interaction for the global expansion of screen cycling. One of the motivations for participating in sports was to seek enjoyment through relationships with people with common goals. Regarding user interaction, the financial success of cycling digitization will significantly change the VR sports market in the future.

## 6. Conclusions

An enhanced version of the planned behavior theory was used in this study to explain the decision-making process of a cyclist who chooses the VR sports application Zwift. The purpose of this study is to examine if the effect of interaction on virtual sports participation has increased because of the COVID-19 pandemic and if there is a difference in decision-making. The importance of social factors may be emphasized even more as a reason for using VR sports apps throughout the protracted COVID-19 outbreak.

Based on literature research, an experimental model was created to characterize the intention to participate in VR sports, and eight associated hypotheses were established. For our analysis, a sample of 301 copies was acquired from a poll of users on Korea’s cycling information sharing website. A multigroup SEM analysis was performed to validate Hypotheses 1–8 using statistical software. Although several hypotheses were not validated, the influence of perceived interaction presented as an extra variable was found to differ depending on the group participating before and after the COVID-19 epidemic, and the study’s goal was achieved.

Only in the group who used Zwift before the COVID-19 outbreak was there a strong influence relationship between perceived behavioral control and intention to use. However, only in the group who used Zwift during the COVID-19 epidemic period was there a significant influence relationship between perceived interactivity and propensity to use. This finding is meaningful for establishing a new trend in VR beyond COVID-19.

This study is significant because it was conducted when the usage of VR sports, which has been identified as a niche market for professional athletes, has been progressively extending to the leisure sports area. Given that information technology has progressed by transcending physical space and socio-cultural barriers to establish a society that links people, the importance of online interaction, such as connection and competitions between users, will be reinforced in VR sports in the future.

This study might highlight some shortcomings in the following areas, insisting the need for more research. For starters, the poll is only available to South Korean consumers. In the future, research that collects data from different nations will yield vital findings in understanding the cultural and national effects of virtual reality sports use. Furthermore, attempts to generalize study findings should be pursued by comparison analysis with high-quality virtual reality sports like screen golf. Second, the study was performed during a period when social distancing measures were tightened as a result of the COVID-19 pandemic, which differs from the present social climate in October 2022. For example, it is required to confirm whether the difference in the influence connection between perceived interactivity and intention to use is still relevant after COVID-19 ends. Third, it is envisaged that additional theories established to describe human behavior would be actively applied in digital sports. The use of the Health Believe Model, 4E (Enable, Encore, Engage, Exemplify), and other models, for example, may lead to a better understanding of the process of adopting digital sports.

## Figures and Tables

**Table 1:** Demographic characteristics of respondents.

Item		n (%)	Item		n (%)
Gender	Male	157 (52.2)	Education Level	Less than high school	4 (1.3)
	Female	144 (47.8)		Attending college	7 (2.3)
Age	Under 20	3 (1.0)		University graduate	283 (94.0)
	20s	44 (14.6)		Over Graduate school	7 (2.3)
	30s	230 (76.4)	Residence	Seoul	74 (24.6)
	40s	18 (6.0)		Busan	30 (10.0)
	50s	6 (2.0)		Daegu	12 (4.0)
Monthly Income	Less than 2 million won	1 (0.3)		Incheon	32 (10.6)
	2~3 million won	4 (1.3)		Gwangju	21 (7.0)
	3~4 million won	27 (9.0)		Daejeon	21 (7.0)
	4~5 million won	15 (5.0)		Ulsan	11 (3.7)
	Over than 5 million won	254 (84.4)		Gyeonggi-do	54 (17.9)
Occupation	Self-employed	84 (27.9)		Gangwon-do	1 (0.3)
	Professional	69 (22.9)		Chungnam-do	9 (3.0)
	Government officer	12 (4.0)		Chungbuk-do	13 (4.3)
	Agriculture/Livestock/Fishery	1 (0.3)		Jeonnam-do	4 (1.3)
	Student	8 (2.7)		Jeonbuk-do	5 (1.7)
	Officer worker	126 (41.9)		Gyeongnam-do	2 (0.7)
	Other	1 (0.3)		Gyeongbuk-do	7 (2.3)
				Jeju	1 (0.3)
				Sejong	4 (1.3)

**Table 2:** Results of confirmatory factor analysis.

Factor	Items	S.E.	t	Standardized Coefficients	C.R.	AVE
Attitudes	To use Zwift is a valuable.	0.083	12.490 ***	0.770	0.911	0.671
	To use Zwift is significant act	0.086	11.329 ***	0.699		
	To use Zwift is worthwhile.	0.085	11.003 ***	0.674		
	To use Zwift is dynamic act.	0.087	11.878 ***	0.729		
	To use Zwift is attractive act.	-	-	0.712		
Subjective Norms	My family members think positive that I use a Zwift.	0.087	10.687 ***	0.731	0.913	0.637
	My friends think positive that I use a Zwift.	0.069	11.771 ***	0.696		
	People around me think positive that I use a Zwift.	0.070	11.716 ***	0.693		
	My family members support that I use a Zwift.	0.083	10.120 ***	0.608		
	My friends support that I use a Zwift.	-	-	0.739		
	People around me support that I use a Zwift.	0.077	12.890 ***	0.767		
Perceived Behavioral Controls	I can use a Zwift whenever I want.	0.090	9.576 ***	0.633	0.864	0.560
	I have enough resources(money) to use a Zwift.	0.096	9.962 ***	0.662		
	I have enough time to use a Zwift.	0.102	9.227 ***	0.607		
	It is easy for me to learn the skills needed to use a Zwift.	0.101	10.319 ***	0.690		
	I can easily obtain the information I need to enjoy Zwift.	-	-	0.687		
Perceived Interactivity	I feel like I am competing with other cyclists.	0.105	10.024 ***	0.724	0.847	0.582
	I feel like I am working out with other cyclists.	0.102	10.336 ***	0.748		
	I feel a sense of fellowship with other cyclists.	-	-	0.664		
	I feel like I am in one place with people who are interested in Zwift.	0.123	10.763 ***	0.817		
Intention to Use	I have a plan to use a Zwift.	0.081	11.969 ***	0.743	0.859	0.670
	I will try to use a Zwift in a long term.	0.086	12.186 ***	0.758		
	I will certainly invest money and time to have a Zwift.	-	-	0.725		

Note: *** *p* < 0.001.

**Table 3:** Results of correlation analysis.

Factor	Attitudes	Subjective Norms	Perceived Behavioral Controls	Perceived Interactivity	Intention to Use	AVE
Attitudes	1					0.671
Subjective Norms	0.789 (0.622)	1				0.637
Perceived Behavioral Controls	0.739 (0.546)	0.693 (0.480)	1			0.560
Perceived Interactivity	0.122 (0.015)	0.580 (0.336)	0.652 (0.425)	1		0.582
Intention to Use	0.774 (0.599)	0.301 (0.091)	0.318 (0.101)	0.363 (0.132)	1	0.670

Note: ( ) denotes the square of the correlation coefficient.

**Table 4:** Summary of tested hypotheses.

Hypothesized Path		Standardized Coefficients	Standard Error	t	Results
H1	Attitudes → Intention to use	0.582	0.186	3.452 **	supported
H2	Subjective Norm → Intention to use	0.130	0.151	0.828	not supported
H3	Perceived Behavioral Control → Intention to use	0.113	0.042	2.343 *	supported
H4	Perceived Interactivity → Intention to use	0.252	0.107	2.564 *	supported

Notes: *: <0.05 **: <0.01.

**Table 5:** Measurement uniformity analysis results.

Model	χ2	df	CFI	RMSEA	TLI	Δχ^2^	Sig.
Unconstrained *	572.79	430	0.955	0.033	0.947	-	
Measurement weights **	586.06	448	0.957	0.032	0.951	Δχ^2^(18) = 13.27 ***	not Sig.

Note: * Unconstrained model: Models without any constraints. ** Measurement weights model: A model with same constraints on group factor loadings. *** χ2 distribution table (*p* = 0.05), χ2(18) = 28.87.

**Table 6:** Multigroup path analysis results.

Path Patterns	Users Who Started during the COVID-19 Pandemic			Users Who Started before the COVID-19 Pandemic		
	Standardized Coefficient	Standard Error	t	Standardized Coefficient	Standard Error	t
Attitudes → Intention to use	0.581	0.260	2.603 **	0.720	0.261	2.802 **
Subjective Norm → Intention to use	0.155	0.217	0.758	0.030	0.206	0.126
Perceived Behavioral Control → Intention to use	0.192	0.206	1.127	0.253	0.117	2.060 *
Perceived Interactivity → Intention to use	0.203	0.073	2.726 **	0.004	0.050	0.052

Note: *: <0.05 **: <0.01.

**Table 7:** Verification results of Hypotheses 5–8 from multigroup SEM analysis.

Hypothesized Path		χ^2^	df	Δχ^2^	Sig
Unconstrained model		572.79	430	-	-
H5	Attitudes → Intention to use	572.81	431	Δχ^2^(1) = 0.02	not Sig.
H6	Subjective Norm → Intention to use	572.99	431	Δχ^2^(1) = 0.20	not Sig.
H7	Perceived Behavioral Control → Intention to use	572.79	431	Δχ^2^(1) = 0.00	not Sig.
H8	Perceived Interactivity → Intention to use	577.94	431	Δχ^2^(1) = 5.15	Sig.

Note: χ^2^ difference test: Δχ^2^(1) = 3.84 (χ^2^ distribution table, *p* = 0.05).

## Data Availability

Not applicable.
